# Relationship between Healthy Lifestyle and Sociodemographic Factors in Adolescents in Catalonia: Application of VISA-TEEN Questionnaire

**DOI:** 10.1371/journal.pone.0163381

**Published:** 2016-09-29

**Authors:** Lluís Costa-Tutusaus, Myriam Guerra-Balic

**Affiliations:** 1 Blanquerna School of Health Science, Ramon Llull University, Barcelona, Spain; 2 Research Group on Health, Physical Activity and Sport (SAFE), Ramon Llull University, Barcelona, Spain; 3 Faculty of Psychology, Education and Sport Sciences, Ramon Llull University, Barcelona, Spain; Institute for Health & the Environment, UNITED STATES

## Abstract

**Introduction:**

There is a clear relationship between the way of life and the health of individuals, and therefore, we can speak of healthy and unhealthy lifestyles. There are different surveys and questionnaires that evaluate the lifestyles of adolescents, but none of them offers a final score that can quantify the healthfulness of an adolescent’s lifestyle. It was with this goal that the VISA-TEEN questionnaire is developed and validated. The objective of this study is to apply the questionnaire to a sample of adolescents who attend school in Catalonia to evaluate the healthfulness of their lifestyles and to relate the scores obtained to different sociodemographic variables.

**Methods:**

Cross-sectional study. A total of 2,832 students from 25 schools in Catalonia responded to the questionnaire. A descriptive analysis was performed, calculating the mean (Standard deviation), median (p_25_, p_75_), and confidence interval. The results were calculated for the total population, factoring according to gender, age, urban/rural population, origin (native/immigrant), and family wealth, which was based on the Family Affluence Scale (FAS II). The significance of the difference was calculated for each factor with the appropriate statistical test.

**Results:**

For the total score of healthy lifestyle, the youngest students and those with the highest family wealth obtained higher scores. With respect to eating habits, girls scored higher than boys, and higher scores were observed in natives and those with high family wealth. For physical activity, boys scored higher, as well as younger individuals, natives, and those from rural areas. With respect to substance abuse, the worst scores were found in older individuals, students from rural areas, and natives. The rational use of leisure technology was only associated with age (worsening scores with older age). Lastly, hygiene was better with girls, decreased with age, and was worse with natives than immigrants.

## Introduction

There is a clear relationship between the way of life and the health of individuals, and therefore, we can speak of healthy and unhealthy lifestyles. The World Health Organization (WHO) defines lifestyle as the way of living based on identifiable patterns or behaviors that are determined by the interaction between individual and personal characteristics, social relationships, and socioeconomic and environmental factors. [1]. With respect to adolescents, the WHO, in its Health Behaviour in School-aged Children (HBSC) survey, considers behaviors related to diet, physical activity, rest, addictions, injuries, hygiene, and sexuality for the lifestyle analysis [2].

There is evidence with respect to how and how much the behaviors evaluated in the HBSC, i.e., diet [3–11], physical activity [12–18], rest [19–26], addictions [27–39], personal hygiene [40–43], and screen-time [44] influence the health of adolescents.

There are different surveys and questionnaires that evaluate the lifestyles of adolescents that are periodically used at the national or supranational level to monitor the habits of this population, and thus, plan health prevention and promotion policies. The WHO promotes the HBSC, a questionnaire that gathers data from 43 countries relative to different areas including, amongst others, nutrition and diet, substance abuse, and sedentary activities in adolescents [45]. The FRESC survey [46] monitors the tendencies of the main risk factors of adolescents in Barcelona and compiles information on the same areas that the HBSC does. Other population studies focus their attention on concrete areas related to lifestyle. The European School Survey Project on Alcohol and other Drugs (ESPAD) gathers information every 5 years regarding tobacco and drug use in adolescents from the 26 countries of the European Union [47]. The Healthy Lifestyle in Europe by Nutrition in Adolescence (HELENA) exploits data gathered with respect to nutrition and physical activity in adolescents from 10 European countries [10, 11, 23, 48, 49]. The National Survey of Child and Adolescent Nutrition (in Spanish “Encuesta Nacional de Alimentación en la población Infantil y Adolescente: ENALIA”) evaluates the nutrition of children and adolescents in Spain [50]. However, these surveys are limited to making percentage-wise evaluations of the responses of each one of the proposed items, and none of them offers a total score that allows for the quantification of the lifestyle healthfulness of adolescents.

For this reason, the VISA-TEEN is developed and validated to evaluate the healthfulness of the lifestyles of adolescents [51]. The questionnaire offers a total score for lifestyle healthfulness and scores on the following 5 components: diet, physical activity, substance abuse, Rational Use of Leisure Technology (RULT), and hygiene. The total score ranges from 0 to 45 points (from least healthy to most healthy), and the scores of the different components range from 0 to 3 points (least healthy to most healthy). The questionnaire offers an acceptable reliability for population studies (α = 0.66; α_est_ = 0.77) and a very good test-retest correlation (CCI = 0.860).

The objective of this study is to apply the questionnaire to a sample of adolescents who attend school in Catalonia to evaluate the healthfulness of their lifestyles and to correlate the scores obtained with the following socio-demographic variables: age, gender, origin (native or immigrant), family wealth, and urban/rural residence.

## Methods

### Sample

The units of analysis were secondary education schools/institutes and vocational training colleges of Catalonia. The 1170 schools that provide these courses were used to define the sample size. Table 1 shows the number of recruited schools stratified by province and ownership. This sample size allowed us to estimate the total score with a precision of ±2 points and with a 95% confidence interval. To ensure the feasibility of the study and the number of schools in each stratum, nonprobability convenience sampling was conducted. Recruiting was performed based on contacts facilitated by the Department of Education of the Catalan government and the education delegations of the different religious congregations (Salesianum, Jesuit, and Gabrielistes). The directors of the centers who agreed to participate signed the corresponding informed consent form and decided, based on internal organizational criteria, what class groups would be surveyed.

**Table 1:** Stratified sampling of the schools based on ownership and province.

	Public schools	% of the total of schools in Catalonia	n of the public sample	Private schools	% of the total schools in Catalonia	n of the private sample
Barcelona	370	**32**	8	499	**43**	11
Girona	70	**6**	1	34	**3**	1
Lleida	53	**5**	1	26	**2**	1
Tarragona	81	**7**	2	37	**3**	1
Total	574		12	596		13

The researcher and two interviewers trained for this purpose visited the schools to present the objectives of the study and to distribute the questionnaires. It was stressed that the questionnaire was anonymous and voluntary. Lastly, a total of 2,832 students completed the questionnaire.

Each school was offered the option of receiving its own results in addition to the global study results.

### Data analyses

The data gathered were entered into the SPSS 20.0 (IBM, Chicago, Illinois, U.S.) for subsequent analysis. The total and factorial scores, Cronbach’s α, and stratified α (which indicates when the scales have more than one dimension) [52–54] were calculated to confirm reliability. The factorial scores (of each component) were calculated with the average of the scores from each item that composed them. A descriptive analysis was conducted to calculate the mean (standard deviation), median (p_25_, p_75_), and their corresponding confidence interval. The results were calculated for the total population, factoring according to gender, age, urban/rural location, origin (native/immigrant), and family wealth, which was based on the Family Affluence Scale (FAS II). The significance of the difference was verified for each factor using the appropriate statistical test, i.e., t-Test or analysis of variance (ANOVA), according to the number of categories of the factor. The association between the scores and age was evaluated with Pearson’s r linear correlation coefficient. The magnitude of the effect was calculated with Cohen’s d in all cases in which significant differences were detected to measure those differences. To interpret the magnitude of the effect, the values proposed by Cohen [55] and expanded by Rosenthal [56] were considered: values between 0.2 and 0.5 imply a small value, between 0.5 and 0.8 indicate a moderate value, between 0.8 and 1.3 indicate a large value, and values greater than 1.3 indicate a very large value. The significance level was 0.05.

### Ethics

Ethics approval was obtained from the Research Ethics Committee (Faculty of Psychology, Education and Sport Sciences, University Ramon Llull). The study was conducted in accordance with the tenets of the Declaration of Helsinki. This study was a self-administrated questionnaire, and at the end, the decision to answer it was from the own students. The school accepted to participate in the project, and the Head of the institution signed the consent. The questionnaire was anonymous, and we guaranteed that the data and the results were going to be totally confidential, and they would only be used globally (the sample of all the schools), not to evaluate particularly each student. The confidentiality was also maintained during data analysis by delinking questionnaire data from any personal identification information. For those over 16, It was not required an informed consent, as it was considered that if students answered the questionnaire, implied an informed acceptance to participate in the study. For those under 16, the researchers did not require parental consent and the principal’s blessing and signature were regarded as sufficient, given that Catalonia school boards and principals should inform parents and collect their consent regarding any extracurricular activity. The research ethics committee also confirmed that the Head’s signature covered the permission to analyse the data of each class group. The Research Ethics Committee approved specifically the informed consent procedure for all participants in our study.

## Results

Of the 2,832 students, 1,717 (60.6%) were students in secondary education, 882 (31.1%) were bachelor’s students, and 233 (8.3%) were vocational students. Table 2 summarizes the socio-demographic characteristics of the sample. The α and stratified α were 0.61 and 0.69, respectively.

**Table 2:** Socio-demographic characteristics of the sample.

	n (%) or $\bar{x}$ (SD)
**Gender**	
Boy	1,208 (50.1)
Girl	1,202 (49.9)
**Age**	
Mean (SD)	15.26 (1.5)
Boy	15.32 (1.53)
Girl	15.21 (1.47)
**Adolescent Origin**	
Native	2,087 (86.7)
Immigrant	319 (13.3)
**Parent origin**	
Both native	1,910 (79.8)
Mixed	128 (5.3)
Both immigrant	354 (14.7)
**School ownership**	
Public	1736 (72)
Private	674 (28)
**Family wealth (FAS II)**	
Low	37 (1.6)
Middle	535 (22.4)
High	1,813 (76)

### Descriptive analysis of the total scores

The mean (SD) of the score was 33.6 (5.5) points, and the median (p_25_-p_75_) was 34 (30–38) points. No significant differences were observed in the total score with respect to gender, urban/rural location, and origin of the adolescent (native/immigrant). However, significant differences were observed in the total score with respect to age and family wealth. With respect to age, the score decreased significantly as age increased (r = -0.391, p<0.01), and the ANOVA test confirmed that there were significant differences between the different ages (p<0.001). S1 Fig shows the mean of the scores and 95% CI for each age.

With respect to family wealth, the score was 2.35 points greater at the high level than at the low level and 1.03 points greater at the high level than at the middle level. The difference between the low and middle levels was 1.31 points. The ANOVA test was significant (p<0.001), and post-hoc analysis showed that the significance could be found between the high and middle levels and between the high and low levels.

Table 3 shows the descriptive statistics and level of significance of all analyses with the total score.

**Table 3:** Descriptive statistics of the total score with respect to the different factors.

	$\bar{x}$ (SD)	95% CI			p	Cohen’s d
**Gender**						
Boy	33.41 (5.57)	33.09	-	33.74	n.s.^1^	
Girl	33.80 (5.51)	33.48	-	34.13		
**Age**						
13	36.54 (4.80)	36.01	-	37.06		
14	35.65 (4.58)	35.23	-	36.08		
15	34.35 (5.00)	33.92	-	34.79	< 0.001^2^	1.49^3^
16	32.20 (5.34)	31.70	-	32.69		
17	30.80 (5.35)	30.27	-	31.34		
18	30.23 (6.04)	29.02	-	31.43		
19	28.81 (6.35)	26.72	-	30.90		
**Location**						
Urban	33.64 (6.13)	32.66	-	34.61	n.s.^1^	
Rural	33.61 (5.50)	33.37	-	33.84		
**Origin**						
Native	33.57 (5.59)	33.32	-	33.82	n.s.^1^	
Immigrant	33.88 (5.21)	33.28	-	34.48		
**Family wealth**						
Low	31.53 (6.18)	29.30	-	33.76		
Middle	32.84 (5.89)	32.33	-	33.36	< 0.001^2^	0.40^4^
High	33.88 (5.40)	33.62	-	34.13		

^1^ significance calculated with Student’s t-test;

^2^ significance calculated with the ANOVA test;

^3^Magnitude of the effect between 13 and 19 years of age;

^4^Magnitude of the effect between high and low levels;

n.s. = not significant

### Descriptive analysis of the scores for each component

#### Diet

The mean (SD) of the score for the diet component was 2.04 (0.5) points, and the median (p_25_-p_75_) was 2 (1.66–2.33) points. No significant differences in diet were observed with respect to urban/rural location. However, significant differences were observed with respect to gender, age, origin (native/immigrant), and family wealth. Girls scored 0.32 points higher than boys (p<0.001). With respect to age, the ANOVA test showed significant differences between the different ages (p<0.01). At 13 years of age, the score was 0.22 points higher than at 19 years of age. With respect to origin, the score was 0.17 points higher for natives than for immigrants. With respect to family wealth, the score was 0.33 points greater at the high level than at the low level and 0.10 points greater at the high level than at the middle level. Table 4 shows the descriptive data, significance, and magnitude of the effect of diet analyses.

**Table 4:** Descriptive analysis of the diet component with respect to the different factors.

	$\bar{x}$ (SD)	95% CI			P	Cohen’s d
**Gender**						
Boy	1.88 (0.46)	1.85	-	1.91		
Girl	2.20 (0.49)	2.17	-	2.22	< 0.001^1^	0.67
**Age**						
13	2.11 (0.49)	2.06	-	2.17	< 0.01^2^	0.46^3^
14	2.04 (0.50)	1.99	-	2.08		
15	2.07 (0.49)	2.03	-	2.11		
16	1.99 (0.52)	1.94	-	2.03		
17	2.03 (0.51)	1.98	-	2.08		
18	1.97 (0.50)	1.87	-	2.06		
19	1.89 (0.45)	1.75	-	2.03		
**Location**						
Urban	2.04 (0.50)	2.02	-	2.06	n.s.^1^	
Rural	2.03 (0.51)	1.94	-	2.12		
**Origin**						
Native	2.06 (0.50)	2.04	-	2.08	< 0.001^1^	0.32
Immigrant	1.89 (0.53)	1.83	-	1.95		
**Family wealth**						
Low	1.74 (0.58)	1.54	-	1.93		
Middle	1/96 (0.52)	1.93	-	2.01	< 0.001^2^	0.61^4^
High	2.07 (0.49)	2.04	-	2.09		

^1^ significance calculated with Student’s t-test;

^2^ significance calculated with the ANOVA test;

^3^Magnitude of the effect between 13 and 19 years of age;

^4^Magnitude of the effect between high and low levels;

n.s. = not significant

#### Physical activity

The mean (SD) of the score for the physical activity component was 2.29 (0.85) points, and the median (p_25_-p_75_) was 2.66 (2–3) points. No significant differences were observed with respect to origin (native/immigrant). However, significant differences were observed with respect to gender, age, location, and family wealth. Boys scored 0.35 points higher than girls (p<0.001). With respect to age, the ANOVA test showed significant differences between the different ages (p<0.01). From 15 years of age, the score decreased as age increased. With respect to origin, the score was 0.17 points higher for natives than for immigrants. With respect to location, the score was 0.24 points higher for those in rural areas (p<0.001). With respect to family wealth, the score was 0.15 points greater at the high level than at the middle level (p<0.01). Table 5 shows the descriptive statistics, significance, and magnitude of the effect of physical activity analyses.

**Table 5:** Descriptive analysis of the physical activity component with respect to the different factors.

	$\bar{x}$ (SD)	95% CI			P	Cohen’s d
**Gender**						
Boy	2.47 (0.76)	2.42	-	2.51	< 0.001^1^	0.42
Girl	2.12 (0.9)	2.06	-	2.17		
**Age**						
13	2.44 (0.75)	2.36	-	2.53	< 0.001^2^	0.66^3^
14	2.46 (0.70)	2.40	-	2.53		
15	2.42 (0.75)	2.36	-	2.49		
16	2.25 (0.85)	2.17	-	2.33		
17	1.97 (1.01)	1.87	-	2.07		
18	2.06 (0.92)	1.88	-	2.24		
19	1.87 (0.96)	1.55	-	2.18		
**Location**						
Urban	2.28 (0.86)	2.24	-	2.31	< 0.001^1^	0.30
Rural	2.52 (0.69)	2.41	-	2.63		
**Origin**						
Native	2.30 (0.84)	2.26	-	2.34	n.s.^1^	
Immigrant	2.26 (0.89)	2.15	-	2.36		
**Family wealth**						
Low	2.12 (0.98)	1.78	-	2.76		
Middle	2.18 (0.92)	2.10	-	2.26	< 0.01^2^	0.23^4^
High	2.33 (0.82)	2.29	-	2.37		

^1^ significance calculated with Student’s t-test;

^2^ significance calculated with the ANOVA test;

^3^Magnitude of the effect between 13 and 19 years of age;

^4^magnitude of the effect between high and low levels;

n.s. = not significant

#### Substance abuse

The mean (SD) of the substance abuse score was 2.50 (0.69) points, and the median (p_25_-p_75_) was 3 (2.25–3) points. No significant differences were observed in substance abuse with respect to gender or family wealth. However, significant differences were observed with respect to age, location (urban/rural), and origin (native/immigrant). With respect to age, the ANOVA test showed significant differences between the different ages (p<0.001), and the post hoc analyses showed that the score decreased with every year that age increased. With respect to location, the score was 0.20 points higher in urban locations than in rural locations (p<0.01). With respect to origin, the score was 0.18 points higher for immigrants than for natives (p<0.001). Table 6 shows the descriptive statistics, significance, and magnitude of the effect of all substance abuse analyses.

**Table 6:** Descriptive analysis of the substance abuse component with respect to the different factors.

	$\bar{x}$ (SD)	95% CI			P	Cohen’s d
**Gender**						
Boy	2.48 (0.70)	2.44	-	2.52	n.s.^1^	
Girl	2.51 (0.67)	2.47	-	2.55		
**Age**						
13	2.85 (0.43)	2.81	-	2.90	< 0.001^2^	1.41^3^
14	2.75 (0.53)	2.71	-	2.80		
15	2.54 (0.66)	2.48	-	2.59		
16	2.31 (0.73)	2.24	-	2.37		
17	2.20 (0.71)	2.14	-	2.27		
18	2.19 (0.75)	2.05	-	2.34		
19	1.89 (0.86)	1.62	-	2.17		
**Location**						
Urban	2.51 (0.68)	2.48	-	2.54	< 0.01^1^	0.27
Rural	2.31 (0.78)	2.18	-	2.43		
**Origin**						
Native	2.48 (0.70)	2.44	-	2.50	< 0.001^1^	0.27
Immigrant	2.66 (0.60)	2.59	-	2.72		
**Family wealth**						
Low	2.58 (0.71)	2.33	-	2.83	n.s.^2^	
Middle	2.49 (0.70)	2.43	-	2.55		
High	2.50 (0.68)	2.47	-	2.53		

^1^ significance calculated with Student’s t-test;

^2^ significance calculated with the ANOVA test;

^3^Magnitude of the effect between 13 and 19 years of age;

n.s. = not significant

#### Rational use of leisure technology (RULT)

The mean (SD) of the score for the RULT component was 1.95 (0.66) points, and the median (p_25_-p_75_) was 2 (1.33–2.33) points. Significant differences were only observed with respect to age (p<0.001) and not for the other factors. For each year, the score decreased until reaching 17 years, at which point no significant differences were observed. Table 7 shows the descriptive statistics, significance, and magnitude of the effect for all RULT analyses.

**Table 7:** Descriptive analysis of the RULT component with respect to the different factors.

	$\bar{x}$ (SD)	95%CI			P	Cohen’s d
**Gender**						
Boy	1.95 (0.69)	1.91	-	1.99	n.s.^1^	
Girl	1.95 (0.63)	1.91	-	1.99		
**Age**						
13	2.16 (0.66)	2.09	-	2.24	< 0.001^2^	0.66^3^
14	2.10 (0.65)	2.04	-	2.15		
15	2.00 (0.62)	1.95	-	2.06		
16	1.84 (0.63)	1.78	-	1.90		
17	1.75 (0.63)	1.69	-	1.81		
18	1.70 (0.72)	1.56	-	1.83		
19	1.71 (0.72)	1.78	-	1.94		
**Location**						
Urban	1.95 (0.66)	1.92	-	1.98	n.s.^1^	
Rural	1.96 (0.73)	1.85	-	2.08		
**Origin**						
Native	1.96 (0.66)	1.93	-	1.99	n.s.^1^	
Immigrant	1.92 (0.66)	1.84	-	1.99		
**Family wealth**						
Low	1.93 (0.75)	1.68	-	2.17	n.s.^2^	
Middle	1.91 (0.67)	1.85	-	1.96		
High	1.96 (0.65)	1.93	-	1.99		

^1^ significance calculated with Student’s t-test;

^2^ significance calculated with the ANOVA test;

^3^Magnitude of the effect between 13 and 19 years of age;

n.s. = not significant

#### Hygiene

The mean (SD) of the score for the hygiene component was 2.38 (0.63) points, and the median (p_25_-p_75_) was 2.5 (2–3) points. No significant differences in hygiene were observed with respect to location (urban/rural) or family wealth. However, there were significant differences with respect to gender, age, and origin (native/immigrant). Girls scored 0.17 points higher than boys (p<0.001). With respect to age, the ANOVA test showed significant differences between the different ages (p<0.05). The lowest score was seen at 18 years of age. With respect to origin, the score was 0.21 points higher for immigrants than for natives (p<0.001). Table 8 shows the descriptive statistics, significance, and magnitude of the effect for all hygiene analyses.

**Table 8:** Descriptive analysis of the hygiene component with respect to the different factors.

	$\bar{x}$ (SD)	95%CI			P	Cohen’s d
**Gender**						
Boy	2.28 (0.67)	2.25	-	2.32	< 0.001^1^	0.28
Girl	2.46 (0.58)	2.43	-	2.50		
**Age**						
13	2.44 (0.60)	2.37	-	2.50	< 0.05^2^	0.33^3^
14	2.41 (0.60)	2.35	-	2.46		
15	2.35 (0.65)	2.30	-	2.40		
16	2.38 (0.61)	2.33	-	2.44		
17	3.34 (0.67)	2.28	-	2.40		
18	2.22 (0.7)	2.09	-	2.36		
19	2.51 (0.68)	2.29	-	2.72		
**Location**						
Urban	2.37 (0.63)	2.34	-	2.40	n.s.^1^	
Rural	2.43 (0.62)	2.34	-	2.53		
**Origin**						
Native	2.35 (0.64)	2.32	-	2.37	< 0.001^1^	0.35
Immigrant	2.56 (0.55)	2.50	-	2.62		
**Family wealth**						
Low	2.20 (0.76)	1.94	-	2.45	n.s.^2^	
Middle	2.33 (0.67)	2.28	-	2.39		
High	2.39 (0.62)	2.36	-	2.42		

^1^ significance calculated with Student’s t-test;

^2^ significance calculated with the ANOVA test;

^3^Magnitude of the effect between 13 and 19 years of age;

n.s. = not significant

#### Comparative summary of all components and factors

S2 Fig is a summary of the differences found in each analyzed component. Highlighted are the factors that showed significant differences, and the magnitude of the effect and the direction of the observed difference (indicating which category had the highest score or, rather, indicating the tendency) are shown inside each box.

## Discussion

### Analysis of the total VISA-TEEN scores

The goal of the study is to place the adolescents in a continuum, and therefore, no cutoff points are provided. However, some general considerations may be made with respect to the scores, understanding that scores on the VISA-TEEN lower than 22.5 imply a lifestyle that negatively affects the health of the adolescent (the score ranges from 0 to 45 points). Scores higher than 30 (2 points on average for each of the 15 items) may be considered a lifestyle that positively favors health, although interpretations should be made carefully because the case could arise that certain items have high scores while others have low scores.

The majority of adolescents scored between 35 and 40 points, and only a few scored lower than 22.5. If we factor according to the socio-demographic variables, there are significant differences with respect to age and family wealth. With respect to age, there is a drop of almost 8 points between 13 and 19 years of age, with a very large magnitude of the effect (d = 1.49). With respect to family wealth, adolescents at the high levels had scores 2.3 points higher than those at the low levels, with a small magnitude of the effect (d = 0.40). These results are comparable with those obtained by Ramos [57], who correlates the global health of Spanish adolescents with their lifestyle. Ramos concludes that there are significant differences in global health with respect to age and family wealth. With regards to age, that author observes that the magnitude of the effect between 12 and 18 years of age is 1.09, a value lower than that obtained by the VISA-TEEN but that may be considered high [55,56]. With respect to family wealth as evaluated based on the FAS II, he obtains a magnitude of the effect of 0.40, exactly the same as what was obtained by the VISA-TEEN. Thus, we may conclude that age and family wealth are associated with the healthy lifestyles of adolescents, but the age effect is greater.

### Score analysis of the different VISA-TEEN components

The range of the scores on each component is between 0 and 3 points, where 0 is an unhealthy lifestyle and 3 is the closest to the recommendations that favor good health.

#### Diet

In contrast to the VISA-TEEN, other studies that evaluate the quality of the diet do not evaluate the consumption of water and carbonated beverages and do evaluate variables that the VISA-TEEN does not, such as the number of portions or daily breakfast intake. These differences in the gathered information make comparisons difficult, but it may be assessed if the observed tendencies are similar. The mean of the scores for the diet component was 2 points. This value corresponds to habits that may not be considered negative for health but that can be improved. In the studies presented by Ayechu in 2010 [58] and Rodríguez et al. in 2012 [59], the results of the Mediterranean diet’s quality index, KIDMED, in 13- to 16-year-old Spanish adolescents are analyzed. In both cases, the largest group is that categorized as “needing improvement”. Similar studies are obtained from the Spanish state by Kontogianni et al. [60], who conclude that the modal group amongst Greek adolescents is also that of “needs improvement”.

Thus, despite evaluating different aspects, the evaluation of our sample and the evaluations of the other aforementioned studies suggest that improvements are needed with respect to the aspects related to the eating habits of adolescents. However, this suggestion should take into account that the results are not extremely negative.

Upon analyzing the relationship of the diet component with the gender variable, it is confirmed that the score is significantly higher for girls (2.20 points on average) than it is for boys (1.88 points on average). We shall again refer to studies that do not evaluate the same variables for comparison. In those cases, the results contradict those obtained by the VISA-TEEN because the studies [58–60] suggest a poorer diet is observed with girls than with boys. This difference lies in the fact that the KIDMED evaluates, amongst other practices, daily breakfast consumption. This question is associated with a negative score, and the percentage of girls who do not eat breakfast daily is greater than that of boys. The study by Dinzeo et al. in 2013 [61] amongst 18- to 25-year-old individuals evaluates cholesterol, sugar, sodium, fruit, and vegetable intake. In the study, as in the VISA-TEEN, girls have higher scores than boys do.

Older adolescents have lower scores than the younger adolescents. This same association between diet and age is also observed in the HBSC-2010, which analyzes fruit, vegetable, carbonated beverage, and daily breakfast intake [2]. This tendency is also seen in the FRESC-2012 [46] study on Catalan adolescents.

With respect to origin, native adolescents scored higher than immigrants. The 2011 study by Prado et al. [62] obtained similar results when it analyzed the eating habits of adolescents from Madrid with the KIDMED. The scores of natives were higher than those of immigrants, except in the case of the Maghrebians, who other studies had already been shown to have eating habits similar to those of Spanish people, possibly due to their origin from a Mediterranean country [63].

With respect to the positive association between family wealth and diet, other studies also show the same relationship. Vereecken et al. [64] concludes that adolescents at the lowest family wealth levels skip more lunches, and the HBSC-2010 [2] also found that in the Spanish state, the adolescents at the lowest levels ate breakfast fewer days per week, ate fewer fruits and vegetables, ate more sweets, and had a higher intake of sweetened beverages.

#### Physical activity

The results on this component, without taking into account socio-demographic factors, may be considered satisfactory. The mean is 2.29 points, and the majority of adolescents (56.61%) scored between 2.5 and 3 points. The inclusion of physical education in the secondary educational curriculum plays an important role in this good score [65]. That the score decreases significantly after 16 years of age, at which time secondary education ends, stands as proof of this assertion.

When physical activity is analyzed with respect to gender, we observe that the score is significantly higher for boys than for girls. Similarly, the analysis of the HELENA-2009 [48] and the HBSC-2010 [2] studies shows that boys have more hours of daily moderate-to-vigorous physical activity than girls do. Other more objective studies, based on measurements taken with an accelerometer, conclude that sedentary lifestyle is greater in girls [66, 67].

Age is also a determining factor in this component. There is a decrease of 0.57 points between the youngest and oldest adolescents, with a moderate magnitude of the effect (d = 0.66). This tendency towards a decrease in physical activity with increased age is also observed in the abovementioned studies, i.e., HELENA-2009 [48] and HBSC-2010 [2]. In 2013, Mitchell et al. published a study [68], based on accelerometer measurements, in which they obtained results in the same direction. The daily median dedicated to moderate or vigorous physical activity decreased from 38.4 minutes at 12 years of age to 27.6 minutes at 15 years of age.

Ortega et al. arrived at the same conclusion in a cohort study of Estonian and Swedish adolescents, showing that in that population, daily physical activity decreased for every year of growth by 2.5 minutes for boys and 1 minute for girls [69].

Location has also been shown to significantly influence physical activity. In rural settings (less than 10,000 inhabitants), adolescents scored 2.5 points higher than in urban settings. These data are comparable with other studies that evaluate the physical condition of school-aged children, conclude that it is superior in rural areas [70, 71], and obtain magnitudes of the effects similar to those obtained by the VISA-TEEN.

Lastly, the same relationship between physical activity and family wealth, based on the FAS II, is observed by the HBSC-2010 [2], Telama et al. [72], the HELENA-2009 study [49], and Borraccino et al. [73]. When comparing the physical activity of adolescents at the low and high family wealth levels, the magnitude of the effect observed in this last study was d = 0.23 for boys and d = 0.27 for girls, values that are comparable to those obtained in the present study (d = 0.23 with no gender difference).

#### Substance abuse

This component is where a stronger influence from one of the factors is produced. Age is clearly associated with the score obtained, with a magnitude of the effect of d = 1.41. Given that the mean was 2.5 points, the analysis of the global score in this component may thus be deceiving. The perspective changes radically when we analyze the data on the different ages. In this case, the score decreases progressively from 2.85 points at 13 years of age to 1.89 at 19 years of age. This association between age and substance abuse coincides with the conclusions published by the health department of the Government of Catalonia when the results from the ESTUDES-2010 survey for Catalonia were analyzed [74] and also with the conclusions published by the Public Health Agency of Barcelona in its report on the risk factors for secondary education students (FRESC-2012) [46].

Urban/rural location was another factor in which significant differences have been found. The adolescents from rural areas scored lower than those from urban areas (the means are 2.31 and 2.51 points, respectively). These results are comparable with those obtained in 2008 in Andalucía [75], where it was concluded that alcohol consumption and illegal drug use patterns are higher in adolescents from rural areas, while tobacco use had no significant differences.

Lastly, origin was also related to substance abuse. Immigrant adolescents had higher scores than natives did. In 2009, Luengo et al. showed that the group of immigrant adolescents in Spain is not uniform, and they recommended approaching the topic of drug consumption in immigrants by segregating according to country of origin [76]. In our analysis, we did not take this possibility into account, given that the resulting groups would have been too small to reach any conclusions.

Nonetheless, given that the largest group in our study is Latin American, we may compare our results with those obtained by Tortajada et al. in 2008 [77]. Their study concludes that alcohol, cannabis, tobacco, amphetamine, hallucinogen, and tranquilizer use is higher in native adolescents.

#### Rational Use of Leisure technology (RULT)

This component is the least influenced by sociodemographic factors (it only showed a relationship with age), and it is the component on which the adolescents have the lowest scores. The use of new technologies to access the internet and connect with others has become universal in recent years. In the last third of 2014, 2.4 million adolescents used the internet [78]. This generalization of internet use increasingly leads to questioning the meaning of rational use and when problematic, abusive, or addictive use may be discussed [79, 80].

The mean score obtained in this component was 1.95 points, and the largest group was between 1.5 and 2 points (32.75%). More than a quarter of respondents had a score lower than 1.5, i.e., had habits that may negatively affect their health. There was no comparable measure in other studies that evaluated the same component of leisure technology use and hours of sleep. Thus, any comparison made would be incomplete. The results obtained by the FRESC-2012 study showed that 40% of adolescents in Barcelona spent more than 2 hours in front of a computer with no academic purpose [46]. More than 2 hours per day is the value that, according to Tsitsika et al., correlates to a problematic use of the internet [81].

With respect to the relationship between RULT and age, the score decreased significantly from 13 to 17 years of age. These data coincide with the results obtained by the FRSC-2012 [46], which observed that the hours dedicated to using the computer for non-academic purposes during the school week increased with age. A study in Japan with nearly 100,000 adolescents [82] concluded that there was an increase in mobile phone use (to talk as well as to chat) at sleep time with age and showed that this negatively influenced the quality of sleep, which decreased with age.

#### Hygiene

On this component, the adolescents obtained a mean score of 2.38 points. Thus, we can consider that it is satisfactory but could improve. The habits of adolescents are close to the recommendations for the frequency of hand washing and tooth brushing. The good score on dental hygiene may be due to the information campaigns by the Catalan government that started in 2006 [83]. The HBSC-2010 [2] concluded that nearly two-thirds of Spanish adolescents brushed their teeth more than once per day, a value that coincides with that obtained by Artácoz et al. in 2007 in the province of Navarra [84]. The frequency of hand washing in adolescents is not well studied at the Spanish level, but the adolescents surveyed by the VISA-TEEN have lived through the campaigns that started with the inauguration of Global Handwashing Day in 2008 [85]. These campaigns had special repercussions after the Type A flu pandemic arrived in Spain in 2009, and even today, there are still many health portals directed to adolescents and the general population [85–87]. These campaigns have contributed, in our opinion, to the good hygienic habits amongst adolescents in Catalonia.

Place of origin is associated with this component. Immigrant adolescents score higher on this component of the VISA-TEEN than native adolescents do. This fact contradicts the findings of other studies, which conclude that there is an inverse relationship. Ramos [88] concludes that immigrant adolescents in Andalucia wash their hands less, and Molina and Pastor show that immigrant children have worse dental health than do native children [89]. By contrast, the results published by Artácoz et al. [84] are similar to those found in our study and note that immigrants brush their teeth with more frequency than natives do.

Thus, this is a controversial component that should be analyzed in more depth, taking into account the country or area of origin of the immigrants.

Age also shows a relationship with hygiene. Once again, we find that the score decreases as age increases, dropping from 2.35 points at 13 years of age to 2.09 at 18 years of age. This tendency towards a worsening of hygienic habits with age is not seen in the HBSC-2010, which concludes that there is no difference in the frequency of tooth brushing amongst the different age groups, although the data from the HBSC-2010 may be masked by gender, i.e., while the frequency increases in girls, it decreases in boys [2].

Lastly, gender was also a factor associated with hygiene. Boys had lower scores than girls. This relationship was also observed, as noted above, in the HBSC-2010, which concluded that, at both the European level and the Spanish national level, the frequency of tooth brushing was higher for girls than for boys. This relationship may be attributed to girls’ concern with body image [2, 90]. Similarly, the study by Vadiakas et al. of 2,481 12- to 15-year-old Greek adolescents shows that girls had a better level of oral hygiene [91].

In conclusion, we may accept that in general terms, the lifestyles of adolescents in Catalonia are satisfactory but that there are components (such as substance abuse and RULT) that need substantial improvement. It is also established that any study of adolescent lifestyles should take into account the socio-demographic factors that influence it, i.e., age, gender, urban/rural location, origin (immigrant/native), and family wealth level.

## Supporting Information

S1 Fig Total score with respect to age. The red line indicates the general mean.
(TIF)

S2 Fig Differences in the lifestyle components with respect to gender, age, location, origin, and family wealth (FAS II). ^1^ RULT = Rational use of leisure technology; ^2^ FAS II: Family wealth measured based on the Family Affluence Scale II. Highlighted boxes are where significant differences were found. The direction of the differences in the score is shown with the mathematical symbols <, >, or =. Higher scores indicate healthier lifestyles in that component. The magnitude of the effect is also indicated with Cohen’s d value. The score tendency is also shown for the age variable.
(TIFF)

S1 File Rough data used in our study. A supplementary file with all the data has been uploaded in an Excel file, in order to offer guarantee to access data sources.
(XLSX)

S2 File Multivariable analysis. (XLS)

## References

[1] WHO. Health Promotion Glossary Geneva: World Health Organitation; 1997.

[2] Moreno C, Ramos P, Rivera F, Jimenez-Iglesias A, García A. Las conductas relacionadas con la salud y el desarrollo de los adolescentes españoles Resumen del estudio Health Behaviour in School Aged Clidren (HBSC-2010). Madrid: Ministerio de Sanidad, servicios sociales e igualdad; 2012.

[3] Maynard M, Gunnell D, Emmett P, Frankel S, Davey Smith G. Fruit, vegetables, and antioxidants in childhood and risk of adult cancer: the Boyd Orr cohort. J Epidemiol Community Health. 2003;57(3):218–25. 10.1136/jech.57.3.218 12594199 PMC1732406

[4] McGartland C, Robson PJ, Murray L, Cran G, Savage MJ, Watkins D, et al Carbonated soft drink consumption and bone mineral density in adolescence: the Northern Ireland Young Hearts project. J Bone Miner Res Off J Am Soc Bone Miner Res. 2003;18(9):1563–9. 10.1359/jbmr.2003.18.9.1563 12968664

[5] Gonzalez-Gross M, Gomez-Lorente JJ, Valtuena J, Ortiz JC, Melendez A. The “healthy lifestyle guide pyramid” for children and adolescents. Nutr Hosp. 2008;23(2):159–68. 18509897

[6] Libuda L, Alexy U, Remer T, Stehle P, Schoenau E, Kersting M. Association between long-term consumption of soft drinks and variables of bone modeling and remodeling in a sample of healthy German children and adolescents. Am J Clin Nutr. 2008;88(6):1670–7. 10.3945/ajcn.2008.26414 19064530

[7] Verzeletti C, Maes L, Santinello M, Vereecken CA. Soft drink consumption in adolescence: associations with food-related lifestyles and family rules in Belgium Flanders and the Veneto Region of Italy. Eur J Public Health. 2010;20(3):312–7. 10.1093/eurpub/ckp150 19805507

[8] Malik VS, Popkin BM, Bray GA, Despres JP, Willett WC, Hu FB. Sugar-sweetened beverages and risk of metabolic syndrome and type 2 diabetes: a meta-analysis. Diabetes Care. 2010 11;33(11):2477–83. 10.2337/dc10-1079 20693348 PMC2963518

[9] Park S, Sherry B, O’Toole T, Huang Y. Factors associated with low drinking water intake among adolescents: the Florida Youth Physical Activity and Nutrition Survey, 2007. J Am Diet Assoc. 2011;111(8):1211–7. 10.1016/j.jada.2011.05.006 21802569

[10] Rey-Lopez JP, Vicente-Rodriguez G, Repasy J, Mesana MI, Ruiz JR, Ortega FB, et al Food and drink intake during television viewing in adolescents: the Healthy Lifestyle in Europe by Nutrition in Adolescence (HELENA) study. Public Health Nutr. 2011;14(9):1563–9. 10.1017/S1368980011000383 21338558

[11] Diethelm K, Jankovic N, Moreno LA, Huybrechts I, De Henauw S, De Vriendt T, et al Food intake of European adolescents in the light of different food-based dietary guidelines: results of the HELENA (Healthy Lifestyle in Europe by Nutrition in Adolescence) Study. Public Health Nutr. 2012;15(3):386–98. 10.1017/S1368980011001935 21936969

[12] Menschik D, Ahmed S, Alexander MH, Blum RW. Adolescent physical activities as predictors of young adult weight. Arch Pediatr Adolesc Med. 2008;162(1):29–33. 10.1001/archpediatrics.2007.14 18180409

[13] US Department of Health and Human Services. 2008 physical activity guidelines for Americans. Washington, DC: Department of Health and Human Services; 2008.

[14] Manonelles P, Alcaraz J, Álvarez J, Jimenez F, Luengo E, Manuz B, et al La utilidad de la actividad física y de los hábitos adecuados de nutrición como medio de prevención de la obesidad en niños y adolescentes. Documento de consenso de la Federación Española de Medicina del Deporte (FEMEDE). Arch Med del Deport. 2008;XXV(5):333–53.

[15] WHO. Recomendaciones mundiales sobre actividad física para la salud. Geneve: Organización Mundial de la Salud; 2010.

[16] Wen CP, Wai JP, Tsai MK, Yang YC, Cheng TY, Lee MC, et al Minimum amount of physical activity for reduced mortality and extended life expectancy: a prospective cohort study. Lancet. 2011 10 1;378(9798):1244–53. 10.1016/S0140-6736(11)60749-6 21846575

[17] World Health Organization regional office for Europe. Promoting sport and enhancing health in European Union countries: a policy content analysis to support action. Copenhagen: publications WHO regional office for Europe; 2011.

[18] Moore SC, Patel A V, Matthews CE, Berrington de G, Park Y, Katki HA, et al Leisure Time Physical Activity of Moderate to Vigorous Intensity and Mortality: A Large Pooled Cohort Analysis. PLoS Med [Internet]. Public Library of Science; 2012 6;9(11):e1001335 Available: 10.1371/journal.pmed.1001335. 23139642 PMC3491006

[19] Goel N, Rao H, Durmer JS, Dinges DF. Neurocognitive consequences of sleep deprivation. Semin Neurol. 2009 9;29(4):320–339. 10.1055/s-0029-1237117 19742409 PMC3564638

[20] Lumeng JC. Future directions for research on sleep durations in pediatric populations. Sleep. 2010 10;33(10):1281–2. 21061849 10.1093/sleep/33.10.1281 PMC2941413

[21] Axelsson J, Sundelin T, Ingre M, Van Someren EJ, Olsson A, Lekander M. Beauty sleep: experimental study on the perceived health and attractiveness of sleep deprived people. BMJ. 2010;341:c6614 10.1136/bmj.c6614 21156746 PMC3001961

[22] Chien KL, Chen PC, Hsu HC, Su TC, Sung FC, Chen MF, et al Habitual sleep duration and insomnia and the risk of cardiovascular events and all-cause death: report from a community-based cohort. Sleep. 2010 2;33(2):177–84. 20175401 10.1093/sleep/33.2.177 PMC2817905

[23] Garaulet M, Ortega FB, Ruiz JR, Rey-Lopez JP, Beghin L, Manios Y, et al Short sleep duration is associated with increased obesity markers in European adolescents: effect of physical activity and dietary habits. The HELENA study. Int J Obes. 2011 10;35(10):1308–17. 10.1038/ijo.2011.149 21792170

[24] Buxton OM, Cain SW, O’Connor SP, Porter JH, Duffy JF, Wang W, et al Adverse metabolic consequences in humans of prolonged sleep restriction combined with circadian disruption. Sci Transl Med. 2012;4(129):129ra43 10.1126/scitranslmed.3003200 22496545 PMC3678519

[25] Morselli LL, Knutson KL, Mokhlesi B. Sleep and insulin resistance in adolescents. Sleep: Journal of Sleep and Sleep Disorders Research 2012;35(10):1313–1314. 10.5665/sleep.2096 PMC3443753 23024425

[26] Leger D, Beck F, Richard J-B, Godeau E. Total Sleep Time Severely Drops during Adolescence. PLoS One [Internet]. Public Library of Science; 2012;7(10):e45204 Available: 10.1371/journal.pone.0045204. 23082111 PMC3474762

[27] Frazier AL, Fisher L, Camargo CA, Tomeo C, Colditz G. Association of adolescent cigar use with other high-risk behaviors. Pediatrics. 2000;106(2):E26 10.1542/peds.106.2.e26 10920182

[28] Salleras L, Taberner JL. Guia per a la prevenció i el control del tabaquisme des de l’àmbit pediàtric Generalitat de Catalunya. Barcelona: Departament de Sanitat i Seguretat Social. Direcció General de Salut Pública; 2003.

[29] Altarriba FX, Bascones A. Libro blanco sobre la relación entre adolescencia y alcohol en España. Madrid: Fund Alcohol y Sociedad; 2006.

[30] García A. Guia de recomendaciones clínicas: Alcoholismo Dirección General de Calidad e Innovación en Servicios Sanitarios. Astúrias: Govierno de Asturias; 2008.

[31] Ochoa Mangado E, Madoz-Gúrpide A, Vicente Muelas N. Diagnóstico y tratamiento de la dependencia de alcohol. Med Segur Trab. 2009;55(214):26–40.

[32] Klempova D, Sánchez A, Vicente J, Barrio G, Domingo A, Suelves JM, et al Consumo problemático de cannabis en estudiantes españoles de 14–18 años: validación de escalas. Madrid: Ministerio de Sanidad y Política Social; 2009.

[33] Shapiro GK, Buckley-Hunter L. What every adolescent needs to know: cannabis can cause psychosis. J Psychosom Res. 2010;69(6):533–9. 10.1016/j.jpsychores.2010.04.002 21109040

[34] Park SH. Smoking and adolescent health. Korean J Pediatr. 2011 10;54(10):401–4. 10.3345/kjp.2011.54.10.401 22232621 PMC3250592

[35] Casadio P, Fernandes C, Murray RM, Di Forti M. Cannabis use in young people: the risk for schizophrenia. Neurosci Biobehav Rev. 2011;35(8):1779–87. 10.1016/j.neubiorev.2011.04.007 21530584

[36] Degenhardt L, Bucello C, Calabria B, Nelson P, Roberts A, Hall W, et al What data are available on the extent of illicit drug use and dependence globally? Results of four systematic reviews. Drug Alcohol Depend. 2011;117(2–3):85–101. 10.1016/j.drugalcdep.2010.11.032 21377813

[37] Madruga CS, Laranjeira R, Caetano R, Pinsky I, Zaleski M, Ferri CP. Use of licit and illicit substances among adolescents in Brazil: a national survey. Addict Behav. 2012 10;37(10):1171–5. 10.1016/j.addbeh.2012.05.008 22703876

[38] Tsiligianni IG, Vardavas CI, Bouloukaki I, Kosmas E, Verigou E, Kiriakaki M, et al The association between alcohol and tobacco use among elementary and high school students in Crete, Greece. Tob Induc Dis. 2012;10(1):15 10.1186/1617-9625-10-15 23009262 PMC3502126

[39] Puente D, Zabaleta-Del-Olmo E, Pueyo MJ, Salto E, Marsal JR, Bolibar B. Prevalencia y factores asociados al consumo de tabaco en alumnos de enseñanza secundaria de Cataluña. Aten Primaria. 2013 Jun-Jul; 45(6): 315–23. 10.1016/j.aprim.2012.12.007 23411164 PMC6985477

[40] Elena M V. Evaluación del estado de salud bucodental y su relación con estilos de vida saludables en la provincia de Salamanca. Salamanca: Universidad de salamanca; 2008.

[41] Tagliaferro EP, Ambrosano GM, Meneghim Mde C, Pereira AC. Risk indicators and risk predictors of dental caries in schoolchildren. J Appl Oral Sci. 2008;16(6):408–13. 10.1590/S1678-77572008000600010 19082400 PMC4327712

[42] Lau CH, Springston EE, Sohn MW, Mason I, Gadola E, Damitz M, et al Hand hygiene instruction decreases illness-related absenteeism in elementary schools: a prospective cohort study. BMC Pediatr. 2012;12:52 10.1186/1471-2431-12-52 22587432 PMC3470997

[43] Holmberg M. Public health and infections: Health in Sweden: The National Public Health Report 2012. Chapter 15. Scand J Public Health. 2012;40(9 Suppl):275–80. 10.1177/1403494812459613 23238413

[44] Bucksch J, Sigmundova D, Hamrik Z, Troped P, Melkevik O, Ahluwalia N, Borraccino A, Tynjala J, Kalman M, Inchley J. International trends in adolescent Screen-Time behaviors from 2002 to 2010. J Adolesc Health. 2016; 10.1016/j.jadohealth.2015.11.014 26827267

[45] Health Behaviour in School-aged Children (HBSC) Network 2014. Health Behaviour in School-aged Children (HBSC): terms of reference. St Andrews (UK): HBSC International Coordinating Centre; 2014 Available: http://www.hbsc.org/about/HBSC%20ToR.pdf.

[46] Pérez A, García-Continente X, Grup Col·laborador enquesta FRESC 2012. Informe FRESC 2012: 25 anys d’enquestes a adolescents escolaritzats de Barcelona. Barcelona: Agència de Salut Pública de Barcelona; 2013.

[47] Hibell B, Guttormsson u, Ahlström S, Balakireva O, Bjarnason T, Kokkevi A, Kraus L. The 2011 ESPAD Report. Substance Use Among Students in 36 European Countries., Stockholm: The Swedish Council for Information on Alcohol and Other Drugs; 2012.

[48] De Cocker K, Ottevaere C, Sjostrom M, Moreno LA, Warnberg J, Valtuena J, et al Self-reported physical activity in European adolescents: results from the HELENA (Healthy Lifestyle in Europe by Nutrition in Adolescence) study. Public Health Nutr. 2011 2;14(2):246–54. 10.1017/S1368980010000558 20236565

[49] Jimenez Pavon D, Ortega FP, Ruiz JR, Espana Romero V, Garcia Artero E, Moliner Urdiales D. Socioeconomic status influences physical fitness in European adolescents independently of body fat and physical activity: the HELENA study. Nutr Hosp. 2010 Mar-Apr;25(2):311–316. 20449543

[50] Agencia Española de consumo, seguridad alimentaria y nutrición. ENALIA [web]. Available: http://www.aecosan.msssi.gob.es/AECOSAN/web/seguridad_alimentaria/subdetalle/enalia.shtml.

[51] Costa-Tutusaus Ll, Guerra-Balic M. Development and psychometric validation of a scoring questionnaire to assess healthy lifestyles among adolescents in Catalonia. BMC Public Health. 2015; 16: 89 Available: http://bmcpublichealth.biomedcentral.com/articles/10.1186/s12889-016-2778-6 . 26821644 10.1186/s12889-016-2778-6 PMC4731967

[52] Cronbach LJ, Schönemann P, McKie D. Alpha coefficients for Stratified-Parallel Tests. Educational and Psychological Measurement 1965;25(2):291–312. 10.1177/001316446502500201

[53] Feldt LS, Brennan RL. Reliability. Whasington D.C: American Council on Education; 1989 p. 105–146.

[54] Tenenbaum G, Eklund RC, Kamata A. Introduction to measurement in sport and exercise psychology In: Tenenbaum G, Eklund RC, Kamata A, eds. Measurement in sport and exercise psychology. Champaign, IL, US: Human Kinetics; 2012 p. 3–7.

[55] Cohen J. Statistical power analysis for the behavioral sciences. 2a ed Hillsdale, NJ, England: Lawrence Erlbaum Associates, Inc; 1977.

[56] Rosenthal JA. Qualitative descriptors of strength of association and effect size. Journal of Social Service Research 1996;21(4):37–59. 10.1300/J079v21n04_02

[57] Ramos P. [Thesis]. Estilos de vida y salud en la adolescencia. Sevilla: Universidad de Sevilla; 2010.

[58] Ayechu A, Durà T. Calidad de los hábitos alimentarios (adherencia a la dieta mediterránea) en los alumnos de educación secundaria obligatoria. An Sist Sanit Navar. 2010;33(1):35–42. 20463769

[59] Rodríguez M, García A, Salinero JJ, Pérez B, Sánchez JJ, Gracia R, et al Calidad de la dieta y su relación con el IMC y el sexo en adolescentes. Nutr clín diet hosp. 2012;32(2):21–27.

[60] Kontogianni MD, Vidra N, Farmaki AE, Koinaki S, Belogianni K, Sofrona S, et al Adherence rates to the Mediterranean diet are low in a representative sample of Greek children and adolescents. J Nutr. 2008 10;138(10):1951–1956. 18806106 10.1093/jn/138.10.1951

[61] Dinzeo TJ, Thayasivam U, Sledjeski EM. The Development of the Lifestyle and Habits Questionnaire-Brief Version: Relationship to Quality of Life and Stress in College Students. Prev Sci. 2013 2 19 10.1007/s11121-013-0370-1 23417669

[62] Prado C, Roville-Sausse F, Marrodan D, Muñoz B, del Olmo RF, Calabria V. Situación somatofisiológica y nutricional de los jóvenes inmigrantes en España. Variación según género y procedencia. Arch Latinoam Nutr. 2011 12;61(4):367–375. 23094519

[63] Mora AI, López-Ejeda N, Anzid K, Montero P, Marrodán MD, Cherkaoui M. Influencia de la migración en el estado nutricional y comportamiento alimentario de adolescentes marroquíes residentes en Madrid (España). Nutr clín diet hosp. 2012;32(supl. 2):48–54.

[64] Vereecken C, Dupuy M, Rasmussen M, Kelly C, Nansel TR, Al Sabbah H, et al Breakfast consumption and its socio-demographic and lifestyle correlates in schoolchildren in 41 countries participating in the HBSC study. Int J Public Health. 2009 9;54 Suppl 2:180–190. 10.1007/s00038-009-5409-5 19639257 PMC3408388

[65] Direcció General de l'Educació Bàsica i el Batxillerat, Generalitat de Catalunya, Departament d'Educació. Orientacions per al desplegament del currículum Educació Física a l'ESO. Març de 2010 [Internet]. Barcelona: Generalitat de catalunya; 2010 [retrieved: 4/04/2016]. Available: http://www.xtec.cat/alfresco/d/d/workspace/SpacesStore/d699dbcd-952b-4ef0-943c-579fee09b56f/orientacions_educacio_fisica_eso.pdf.

[66] Colley R, Garriguet D, Janssen I, Wong S, Saunders T, Carson V, et al The association between accelerometer-measured patterns of sedentary time and health risk in children and youth: results from the Canadian Health Measures Survey. BMC Public Health 2013;13(1):200 10.1186/1471-2458-13-200 23497190 PMC3599834

[67] Chung AE, Skinner AC, Steiner MJ, Perrin EM. Physical activity and BMI in a nationally representative sample of children and adolescents. Clin Pediatr. 2012 2;51(2):122–129. 10.1177/0009922811417291 22315503 PMC3368285

[68] Mitchell JA, Pate RR, Beets MW, Nader PR. Time spent in sedentary behavior and changes in childhood BMI: a longitudinal study from ages 9 to 15 years. Int J Obes. 2013 1;37(1):54–60. 10.1038/ijo.2012.41 22430304

[69] Ortega FB, Konstabel K, Pasquali E, Ruiz JR, Hurtig-Wennlof A, Maestu J, et al Objectively measured physical activity and sedentary time during childhood, adolescence and young adulthood: a cohort study. PLoS One 2013 4 23;8(4):e60871 10.1371/journal.pone.0060871 23637772 PMC3634054

[70] De la Cruz-Sánchez E, Aguirre-Gómez M, Pino-Ortega J, Díaz-Suárez A, Valero-Valenzuela A, García-Pallarés J. Diferencias en la condición física en niños de entornos rurales y urbanos. Revista de Psicología del Deporte 2012;21(2):359–363.

[71] Chillon P, Ortega FB, Ferrando JA, Casajus JA. Physical fitness in rural and urban children and adolescents from Spain. J Sci Med Sport. 2011 9;14(5):417–423. 10.1016/j.jsams.2011.04.004 21620767

[72] Telama R, Laakso L, Nupponen H, Rimpela A, Pere L. Secular trends in youth physical activity and parents' socioeconomic status from 1977 to 2005. Pediatr Exerc Sci. 2009 11;21(4):462–474. 10.1123/pes.21.4.462 20128365

[73] Borraccino A, Lemma P, Iannotti RJ, Zambon A, Dalmasso P, Lazzeri G, et al Socioeconomic effects on meeting physical activity guidelines: comparisons among 32 countries. Med Sci Sports Exerc. 2009 4;41(4):749–756. 10.1249/MSS.0b013e3181917722 19276860 PMC2663903

[74] Departament de Salut, Generalitat de catalunya. Informe dels resultats per a Catalunya de l'Enquesta estatal sobre l'ús de drogues a l'ensenyament secundari (ESTUDES) 2010 [Internet]. Barcelona: Generalitat de Catalunya; 2012 [retrieved: 4/4/2016]. Available: http://www20.gencat.cat/docs/canalsalut/Minisite/Drogues/Professionals/Epidemiologia/docs/estudes2010.pdf.

[75] Pérez A, Ramírez EM, Jiménez I, Leal FJ, Martínez ML, Pérez R. Diferencias en el consumo urbano y rural de alcohol, tabaco y drogas en adolescentes. Med fam Andal. 2008;9(1):10–17.

[76] Luengo MA, Villar P, Sobral J, Romero E, Gómez-Fraguela JA. El consumo de drogas en los adolescentes inmigrantes: implicaciones para la prevención. Revista Española de Drogodependencias 2009;34(4):448–479.

[77] Tortajada S, Valderrama JC, Castellano M, Llorens N, Agulló V, Herzog B, et al Consumo de drogas y su percepción por parte de inmigrantes latinoamericanos. Psicothema 2008;20(3):403–407. 18674434

[78] Ureña A. La sociedad en la red Informe anual 2012. Madrid: Ministerio de Energía, Industria y Turismo; 2013.

[79] Matute H, Vadillo MA. Psicología de las nuevas tecnologías De la adicción a Internet a la convivencia con robots. Madrid: Síntesis; 2012.

[80] Bernabeu J. Usos adolescents de les xarxes socials i d'altres TIC [Internet]. Granollers: Servei de salut pública de l'ajuntament de Granollers; 2011 [Retreived: 4/4/2016]. Available: http://www.slideshare.net/sobredrogues/pantalles-11-9907298.

[81] Tsitsika A, Critselis E, Janikian M, Kormas G, Kafetzis DA. Association between internet gambling and problematic internet use among adolescents. J Gambl Stud. 2011 9;27(3):389–400. 10.1007/s10899-010-9223-z 20953681

[82] Munezawa T, Kaneita Y, Osaki Y, Kanda H, Minowa M, Suzuki K, et al The association between use of mobile phones after lights out and sleep disturbances among Japanese adolescents: a nationwide cross-sectional survey. Sleep 2011 8 1;34(8):1013–1020. 10.5665/SLEEP.1152 21804663 PMC3138156

[83] Casals E, Romero N. Dents fortes i sanes. Glopeig + raspall. Barcelona: Direcció general de Salut Pública; 2006.

[84] Artazcoz J, Martinicorena FJ, Gallardo ER, Rodriguez PG, Bravo M. Percepción y hábitos de salud bucodental en niños y adolescentes de Navarra, 2007. An Sist Sanit Navar. 2010;33(1):51–64. 20463771 10.4321/s1137-66272010000100006

[85] Globalhandwashing.org [Internet]. New York: The Global Public-Private Partnership for Handwashing with Soap (PPPHW); 2013[Retrieved: 4/4/2016]. Available: http://www.globalhandwashing.org/.

[86] UNICEF.es [Internet]. Barcelona: UNICEF; 2011 [Retrieved: 4/4/2016]. Rentar-se les mans amb sabó pot prevenir milions de morts per malalties contagioses. Available: http://www.unicef.es/cat/sala-premsa/rentar-se-les-mans-amb-sabo-pot-prevenir-milions-de-morts-malalties-contagioses.

[87] Faros [Internet]. Barcelona: Sant Joan de Déu; 2013 [Retrieved: 4/4/2016]. Les mans netes eviten malalties i salven vides! Available: http://faros.hsjdbcn.org/ca/articulo/mans-netes-eviten-malalties-salven-vides.

[88] Ramos L.. Hábitos, comportamientos y actitudes de los adolescentes inmigrantes sobre nutrición Recomendaciones educativas. Granada: Universidad de Granada; 2007.

[89] Molina MC, Pastor C. Cos i salut en persones immigrades. Temps d'Educació 2007; 33:49–59.

[90] Currie C, Zanotti C, Morgan A, Currie D, Looze Md, Roberts C, et al Social determinants of health and well-being among young people Health Behaviour in School-aged Children (HBSC) study: international report from the 2009/2010 survey. Copenhagen: World Health Organization Regional Office for Europe; 2012.

[91] Vadiakas G, Oulis CJ, Tsinidou K, Mamai-Homata E, Polychronopoulou A. Oral hygiene and periodontal status of 12 and 15-year-old Greek adolescents. A national pathfinder survey. Eur Arch Paediatr Dent. 2012 2;13(1):11–20. 10.1007/BF03262835 22293100

