# Supplementary figures and images for: Relationship between Healthy Lifestyle and Sociodemographic Factors in Adolescents in Catalonia: Application of VISA-TEEN Questionnaire
Source: PLoS One. 2016 Sep 29;11(9):e0163381. doi: 10.1371/journal.pone.0163381 (PMC5042508; doi:10.1371/journal.pone.0163381)

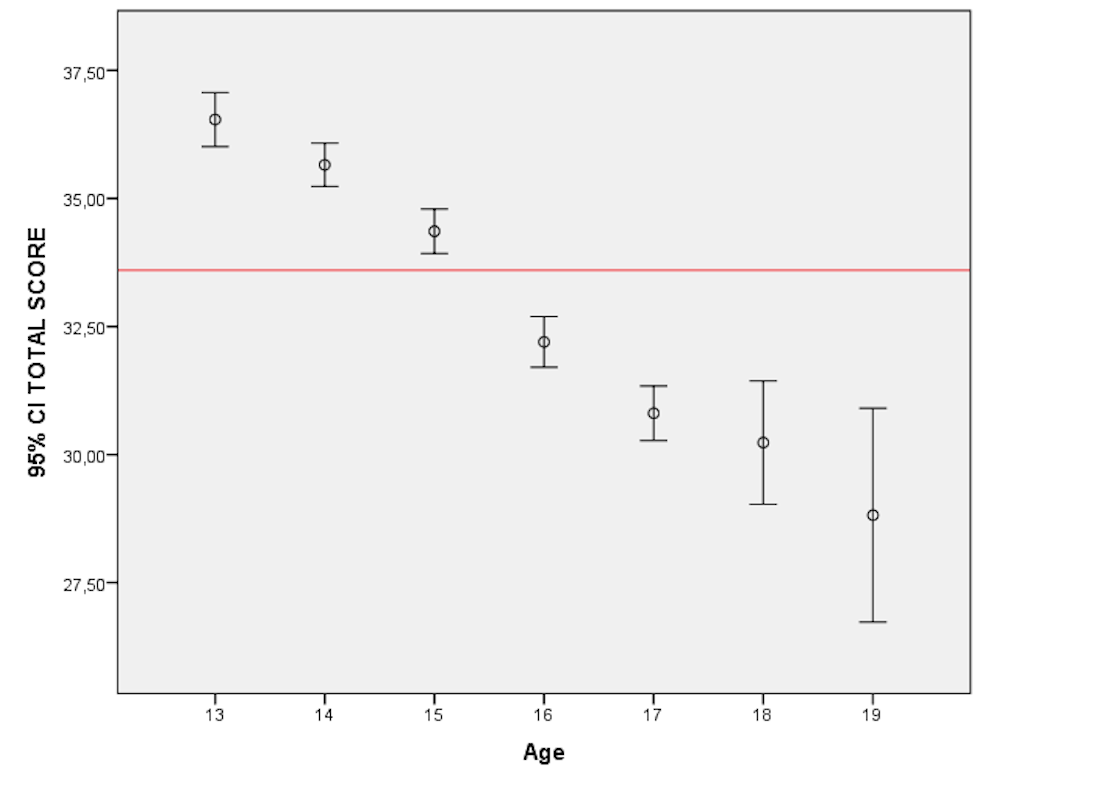

Supplement: S1 Fig — The red line indicates the general mean. (TIF) [file pone.0163381.s001.tif]

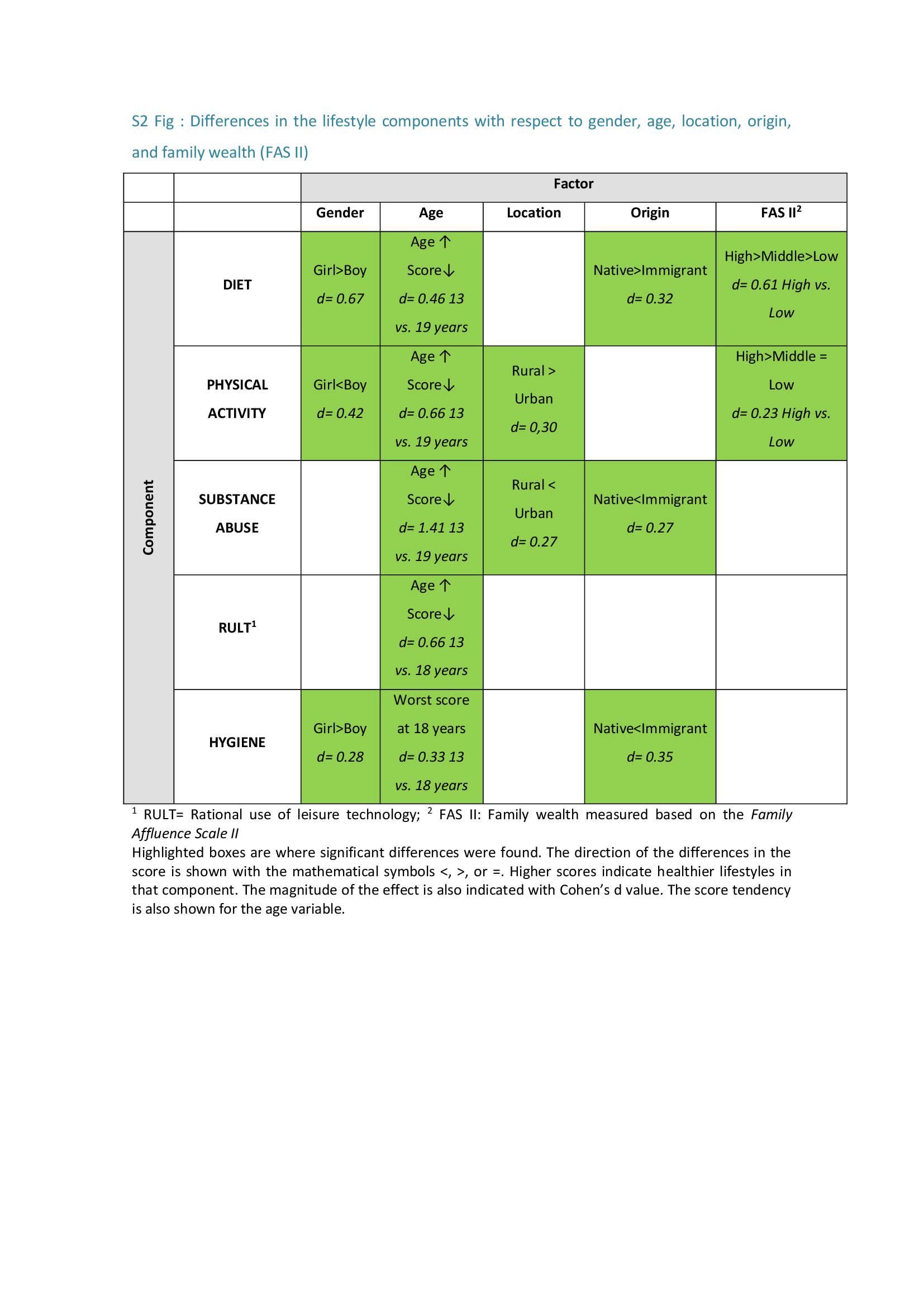

Supplement: S2 Fig — 1 RULT = Rational use of leisure technology; 2 FAS II: Family wealth measured based on the Family Affluence Scale II. Highlighted boxes are where significant differences were found. The direction of the differences in the score is shown with the mathematical symbols <, >, or =. Higher scores indicate healthier lifestyles in that component. The magnitude of the effect is also indicated with Cohen’s d value. The score tendency is also shown for the age variable. (TIFF) [file pone.0163381.s002.tiff]
